# Scar-Free Healing of Endometrium: Tissue-Specific Program of Stromal Cells and Its Induction by Soluble Factors Produced After Damage

**DOI:** 10.3389/fcell.2021.616893

**Published:** 2021-02-25

**Authors:** Roman Eremichev, Maria Kulebyakina, Nataliya Alexandrushkina, Peter Nimiritsky, Nataliya Basalova, Olga Grigorieva, Mane Egiazaryan, Daniyar Dyikanov, Vsevolod Tkachuk, Pavel Makarevich

**Affiliations:** ^1^Medical Research and Education Center, Institute for Regenerative Medicine, Lomonosov Moscow State University, Moscow, Russia; ^2^Faculty of Medicine, Lomonosov Moscow State University, Moscow, Russia; ^3^Laboratory of Molecular Endocrinology, National Medical Research Center of Cardiology, Moscow, Russia

**Keywords:** healing, fibrous tissue, regeneration, myofibroblast, endometrium, menstruation, extracellular matrix, mesenchymal stromal cells

## Abstract

Besides certain exceptions, healing of most tissues in the human body occurs *via* formation of scar tissue, rather than restoration of lost structures. After extensive acute injuries, this phenomenon substantially limits the possibility of lost function recovery and, in case of chronic injury, it leads to pathological remodeling of organs affected. Managing outcomes of damaged tissue repair is one of the main objectives of regenerative medicine. The first priority for reaching it is comparative investigation of mechanisms responsible for complete restoration of damaged tissues and mechanisms of scarring. However, human body tissues that undergo complete scar-free healing are scarce. The endometrium is a unique mucous membrane in the human body that heals without scarring after various injuries, as well as during each menstrual cycle (i.e., up to 400 times during a woman’s life). We hypothesized that absence of scarring during endometrial healing may be associated with tissue-specific features of its stromal cells (SCs) or their microenvironment, since SCs transform into myofibroblasts—the main effector link of scarring. We found that during healing of the endometrium, soluble factors are formed that inhibit the transition of SCs into myofibroblasts. Without influence of these factors, the SCs of the endometrium undergo transformation into myofibroblasts after transforming growth factor β1 (TGF-β1) treatment as well as the SCs from tissues that heal by scarring—skin or fat. However, unlike the latter, endometrial SCs organize extracellular matrix (ECM) in a specific way and are not prone to formation of bulky connective tissue structures. Thus, we may suggest that tissue-specific features of endometrial SCs along with effects of soluble factors secreted *in utero* during menstruation ensure scar-free healing of human endometrium.

## Introduction

Formation and expansion of fibrous tissue known as fibrogenesis occur at the site of injury and in adjacent tissues. In the human body, it is an integral feature of normal physiological response to damage (Kisseleva and Brenner, 2008; Rockey et al., 2015).

Despite its obvious helpfulness from the survival point of view, fibrogenesis poses a challenge for modern healthcare. Human organs undergoing fibrosis after the damage of different kinds are subject to further pathologic remodeling and loss of function resulting in chronic disease. Besides, fibrotic tissue limits the mobility of surrounding tissues and creates an obstacle to the formation of new functional structures at the lesion site. Hence, establishment of molecular mechanisms of fibrogenesis may also be important for the development of methods that prevent fibrosis and stimulate the recovery of normal structures after damage. Its challenging nature and complexity drive multiple studies in the field, and in spite of significant advances, effective prevention of fibrosis in humans is still to be reached (Atala et al., 2010; Wynn and Ramalingam, 2012; Rosenbloom et al., 2017). The fact that fibrogenesis is a part of the evolutionarily established response to damage further complicates the issue. The task is not just “prevention of fibrosis,” which may lead to interruption of healing, but to modulate the healing process and facilitate recovery of damaged tissue with original structure and function.

The human body does have a limited number of structures able to respond to different kinds of injuring events preferably by regeneration. These include the endometrium—the internal mucous layer of the uterus reaching 10–15 mm thickness in adult healthy women. The endometrium has a remarkable ability to regenerate after damage without scarring. This occurs both after surgical scraping of endometrium known as curettage and after its full-thickness section during resectoscopic tumor removals (Johannisson et al., 1981; Deutsch et al., 2017). Moreover, the endometrium heals without fibrogenesis after Cesarean section that requires a lengthy perforating section of the uterine wall or even its minor rupture depending on technique and course of operation (Ben-Nagi et al., 2009). Thus, the endometrium undergoes healing without obvious fibrogenesis (i.e., fibrogenesis is limited or does not occur).

*Homo sapiens* is one of the few species that possess an interesting feature relying on endometrial regeneration. That can be described as a physiological renewal of endometrium *via* its self-damage—namely, menstruation, which swiftly transits to rapid healing without fibrosis and ends up by the full recovery of its structure in every menstrual cycle. The latter is high in number and in healthy women may account for up to 400 episodes of endometrial self-damage and regeneration without scarring.

A comprehensive review of menstruation mechanisms is provided by Maybin and Critchley (2015), and this pool of data is being permanently expanded due to its importance for healthcare and reproductive medicine. However, little is known about mechanisms providing endometrial healing without fibrosis after damage in the menstrual cycle, parturition, or surgery.

We paid attention to stromal cells (SCs) of the endometrium as far as in most tissues, SCs are major effectors of fibrogenesis (Rosenbloom et al., 2017). At the site of injury, they undergo transition to myofibroblasts—cells that directly execute fibrogenesis and are able to contract mediating wound closure and providing mechanical support of the healing tissue (Kisseleva and Brenner, 2008; Hinz, 2016a). The main factors that drive the acquisition of myofibroblast phenotype by SCs include blood clotting, inflammation, and mechanical impact (Hinz, 2016b). Among numerous molecular regulators, it has been established that transforming growth factor β1 (TGF-β1) is sufficient and indispensable for induction of myofibroblast transition in SC, which allowed its use in models of fibrogenesis (Lodyga and Hinz, 2020).

Damaged tissues have a high concentration of TGF-β1: it is released from activated platelets, produced by recruited monocytes and macrophages; eventually, it is secreted by cells of damaged tissue especially under mechanical stress (Barrientos et al., 2008; Hinz et al., 2019). TGF-β1 binds to its receptors, leading to activation of SMAD signaling and SMAD-independent signaling pathways, including mitogen-activated protein kinase (MAPK) pathways and small GTPases such as RhoA. This results in increased expression of genes including myofibroblast markers—α-smooth muscle actin (α-SMA), type I collagen (collagen I), and a splice variant of fibronectin that carries an extracellular domain A (ED-A fibronectin) (Kim et al., 2018).

An increased production and decreased degradation of extracellular matrix (ECM) shift the balance toward its deposition (Rosenbloom et al., 2017). At the same time, stress fibers are rapidly formed *via* polymerization of actin with inclusion of α-SMA providing the high contractile ability of myofibroblasts. Stress fibers are assembled around focal adhesion contacts that mediate interaction of cytoskeleton with ECM *via* talin, vinculin, and a repertoire of integrins (Hinz et al., 2019).

Thus, the impact of TGF-β1 released after damage may result in a stable transition of SCs to myofibroblasts, but due to unknown reasons, this does not seem to occur during the healing of the endometrium (Abberton et al., 1999; Bellofiore et al., 2018). We suggested that it may be caused by a resistance of endometrial SC to factors that induce myofibroblast phenotype acquisition (e.g., TGF-β1). However, endometrial stromal cells (EndoSCs) might also be targeted by stimuli produced during menstruation and these may prevent EndoSC from myofibroblast transition. To test these assumptions, we compared the ability of EndoSC to undergo myofibroblast transition using dermal stromal cell (DermSC) and adipose tissue stromal cell (AdipoSC) as control cells. We also evaluated the influence of soluble factors released during menstruation and contained in menstrual discharge.

## Materials and Methods

### Cell Culture

Isolated SCs were cultured using uncoated culture dishes or plates (TPP, Switzerland) in an appropriate growth medium according to a previously performed experimental selection:

• EndoSCs were cultured in Dulbecco’s modified Eagle’s medium (DMEM)/F12 (Gibco, United States) + 10% fetal bovine serum (FBS; HyClone, United States).
• DermSCs in DMEM low glucose (Gibco, United States) + 10% FBS (HyClone, United States).
• AdipoSCs in AdvanceStem Medium (HyClone, United States) + 10% AdvanceStem Supplement (HyClone, United States).

The medium was changed every 2–4 days, and when confluence reached 80–90%, cells were detached and passaged at 1:3–1:4 ratio. Trypsin-EDTA (0.05%) solution (Gibco, United States) was used for detachment of cells from the culture plastic. Obtained suspension was transferred into a tube with 5 ml of complete growth medium and centrifuged at 200 g for 5 min, then supernatant was removed, and the cells were resuspended in growth medium and seeded to clean culture dishes.

### Isolation of Adipose Stromal Cells

Adipose tissue samples were obtained from three healthy donors chosen from inpatients of Lomonosov Moscow State University Medical Centre. Tissue sample was placed into a tube with Hanks’ balanced salt solution (HBSS; Paneco, Russia) containing 500 U/ml of penicillin and 500 μg/ml of streptomycin (Gibco, United States) and transported to the laboratory. Obtained material was minced to a homogeneous state with sterile surgical scissors under a laminar hood and placed into a tube. The homogenate was then subjected to enzymatic treatment in AdvanceStem Medium (HyClone, United States) with a mixture of collagenase I (200 U/ml, Worthington, United States) and dispase (30 U/ml, Corning, United States) added in a volume equal to the minced tissue suspension. Enzymatic digestion lasted 60 min at 37°C with intense shaking every 5–10 min. After digestion, an equal volume of AdvanceStem Medium (HyClone, United States) with 10% AdvanceStem Supplement (HyClone, United States) was added to the resulting mixture to inactivate enzymes. After subsequent centrifugation at 200 g for 10 min, the supernatant and floating fraction of adipose tissue were discarded and the pellet was resuspended in complete growth medium (AdvanceStem Medium, HyClone, United States) with 10% AdvanceStem Supplement (HyClone, United States). The obtained suspension was filtered through nylon strainers with pore size of 100 μm (BD Falcon, United States) to remove debris. Filtrate was centrifuged (200 g, 5 min), supernatant was removed, and the pellet was resuspended in complete growth medium followed by transfer to an uncoated cell culture dish of a suitable size. After overnight culture, the medium was changed and isolated AdipoSCs were cultured as described above (see section “Cell Culture”).

### Isolation of Dermal Stromal Cells

Human skin samples were obtained from three healthy donors. Every sample was cut with sterile scissors in a laminar box to pieces of no more than 0.25 cm^3^ that were placed into a tube. Equal volume of DMEM low glucose containing a mixture of type I collagenase (300 U/ml, Worthington, United States) and dispase (30 U/ml, Corning, United States) was added to the cut skin. Enzymatic digestion lasted 60 min at 37°C with intense shaking every 5–10 min. After digestion, an equal volume of complete growth medium [DMEM low glucose (Gibco, United States) with 10% FBS (Gibco, United States)] was added to the resulting mixture to inactivate enzymes. Obtained suspension was centrifuged at 200 g for 10 min, and supernatant was removed. The pellet was resuspended in complete growth medium and transferred to a cell culture dish of suitable diameter. The next day, the growth medium was changed and isolated DermSCs were cultured as described above (see section “Cell Culture”).

### Isolation of Endometrial Stromal Cells and Preparation of Menstrual Discharge Serum

Menstrual discharge was collected from healthy volunteer female donors using a menstrual cup (Tulip, Russia) for 6–8 h during the second day of menstruation. Obtained discharge was transferred into a sterile 50 ml tube, and biomaterial was delivered to the laboratory at + 4°C within 2–3 h. Immediately after delivery, menstrual discharge was diluted twofold by serum-free DMEM/F12 (Gibco, United States), gently resuspended and centrifuged for 20 min at 300 g (centrifuge: SL40 R with swinging bucket rotor, Thermo Fisher Scientific, United States; acceleration 9, deceleration 7). The supernatant [menstrual discharge serum (MDS)] was collected with a 25 ml syringe with a needle into a separate 50 ml tube and left until the centrifugation step described below.

The cell pellet was resuspended in four volumes of dilution buffer consisting of 1% FBS (Gibco, United States) in HBSS (Paneco, Russia) with 5 mM ethylenediaminetetraacetic acid (EDTA; Panreac, United States). Then, it was intensively pipetted with a 25 ml serological pipette tip and carefully layered on 13 ml of Ficoll (1.077 g/cm^3^, Paneco, Russia) in a 50 ml tube. The tube was allowed to stand for 10 min for sedimentation of blood clots and large pieces of tissue into the thickness of the Ficoll. Then, the tube was carefully transferred to a centrifuge (SL40 R with swinging bucket rotor, Thermo Scientific, United States) avoiding phase mixing.

Tube containing MDS was placed to the same bucket rotor and centrifuged for 40 min at 500 g (acceleration 9, deceleration 5). After centrifugation, the supernatant from the MDS tube was collected with a 25 ml syringe with needle and filtered through a 0.22 μm filter (TPP, Switzerland), and the obtained liquid was designated as 25% MDS (due to prior twofold menstrual discharge dilution above and initial ratio of cells to serum volume about 1:1).

After centrifugation, 3/4 of the supernatant were discarded. Residual supernatant, cells, and small pieces of tissue were collected from the Ficoll surface into a 15 ml tube using a 10 ml pipette, diluted to a volume of 14 ml by dilution buffer and centrifuged for 10 min at 300 g. Supernatant was discarded, pellet was resuspended in 2 ml of complete growth medium [DMEM/F12 (Gibco, United States) with 10% FBS (HyClone, United States)] and transferred to a 35 mm cell culture dish for 1 h. Thereafter, the excess of sedimented, but non-adhered, cells was detached by gentle manual shaking of the dish, and the medium was changed. After 24 h, the medium was changed again and isolated EndoSCs were cultured as described above (see *Cell Culture*). The cells were successfully isolated from 10 samples, and three cultures were chosen for subsequent experiments.

To assess the quality of the obtained MDS and its usability for cell culture, 500 μl of “25% MDS” was placed in a well of a 48-well plate and was incubated for 24 h at 37°C, while the remaining MDS was frozen in liquid nitrogen and stored at −20°C. After 24 h, the MDS was analyzed by phase contrast microscopy: in the case of protein aggregate formation or germ contamination, frozen MDS sample was utilized. If MDS remained transparent was kept, MDS was deemed appropriate for *in vitro* use. Before the beginning of the experiments, MDS samples obtained from 15 donors were thawed and mixed into one stock solution, which was aliquoted, frozen again in liquid nitrogen, and stored at −80°C until use.

### Isolation of Blood Serum

The venous blood (10–15 ml) was collected into tubes containing coagulation activator (BD Vacutainer, United States) from the cubital vein of 15 healthy volunteer female donors at the second day of menstruation. After completion of blood clotting, tubes were centrifuged for 20 min at 300 g. Blood serum (BS) was collected with a 10 ml syringe with a needle, diluted four times with DMEM/F12 (Gibco, United States), filtered through a 0.22 μm filter (TPP, Switzerland) to comply with MDS isolation protocol, then frozen in liquid nitrogen and stored at −20°C. The resulting liquid was used for further experiments as 25% BS. Before the experiments, BS samples obtained from 15 donors were thawed and mixed into one stock solution, which was aliquoted, frozen again in liquid nitrogen, and stored at −80°C until use.

### Flow Cytometry

For immunophenotyping of SCs, the MSC Phenotyping Kit (130-095-198, Miltenyi Biotec GmbH, Germany) and antibody to CD31 (303124, Biolegend, United States) with corresponding control (400158, Biolegend, United States) were used according to the manufacturer’s instructions. Cells were washed with Versene solution (Paneco, Russia), detached from plastic with 0.05% trypsin-EDTA solution (Gibco, United States), and after trypsin inhibition by complete growth medium, they were aliquoted in four test tubes and centrifuged for 10 min at 300 g. Pellets were then resuspended in flow cytometry buffer [1% bovine serum albumin (BSA; Sigma, United States) in HBSS (Paneco, Russia)] and incubated for 10 min at 4°C with antibodies to CD31 or cocktail of antibodies to CD73, CD90, CD105, CD14, CD20, CD34, CD45 from the kit or corresponding control antibodies at a dilution provided by the manufacturer. After labeling, cells were diluted by 1 ml of flow cytometry buffer, centrifuged for 10 min at 300 g, then supernatant was discarded, and cells were resuspended in 500 μl of flow cytometry buffer. Cells were detected using BD LSR Fortessa flow cytometer (BD Biosciences, United States), and results were analyzed using the FlowJo software (BD, United States).

### Adipogenic and Osteogenic Differentiation of Stromal Cells

Cell differentiation was performed using StemPro Adipogenesis Differentiation Kit (Gibco, United States) and StemPro Osteogenesis Differentiation Kit (Gibco, United States) according to the manufacturer’s instructions. SCs were seeded in 12-well plates at a density of 100,000 cells per well and cultured during 2–3 days until reaching 100% confluency, and growth media have been replaced by differentiation induction media. For adipogenic differentiation, the medium was changed every 2–3 days, and cells were fixed with 4% formaldehyde (Panreac, United States) for 30 min on day 14; lipid droplets were stained with Oil Red O solution from Mesenchymal Stem Cell Adipogenesis Kit (Chemicon, United States) according to the manufacturer’s instructions. For osteogenic differentiation, the medium was changed every 3–4 days, and cells were fixed with 4% formaldehyde for 30 min on day 21; mineral deposits were stained by Alizarin red solution from Mesenchymal Stem Cell Osteogenesis Kit (Chemicon, United States) according to the manufacturer’s instructions. Visualization and image acquisition were performed on an inverted Leica DMi8 microscope with a DFC7000T camera, and images were processed in Fiji package (Schindelin et al., 2012) based on ImageJ (NIH, United States).

### Chondrogenic Differentiation of Stromal Cells

Cell differentiation was performed using StemPro Chondrogenesis Differentiation Kit (Gibco, United States). Pellets consisting of 350 ^∗^ 10^3^ SCs were formed by centrifugation at 300 g for 10 min in 15 ml tubes (Corning, United States). Then, 1.5 ml of differentiation medium from the kit was gently added atop, and the caps of the tubes were replaced with those containing an air filter. Full culture media specific for every SC type were used as control media. After 4 days, pellets transformed to spheroids, and from this point, the media were changed two times a week for 17 days. At the end of the culture period, spheroids were frozen in OCT medium and were sectioned on Leica CM1850 cryotome. Up to 10 sections per 10 microns were obtained from every specimen and were placed on a separate slide. Then, prepared sections were fixed in 4% formaldehyde followed by rinse in PBS for 15 min. Slides with the differentiated cells and slides with the corresponding controls were stained in parallel with Alcian blue (Sigma, United States) according to the following protocol:

1) Incubation in 3% acetic acid for 3 min
2) Incubation in 1% Alcian blue solution for 30 min
3) Washing with an excess of 3% acetic acid (40 dips)
4) Washing in running tap water for 10 min
5) Washing in distilled water (10 dips)
6) Dehydration and clearing: 1 min 70% alcohol, 1 min 96% alcohol, 3 min xylene
7) Embedding with… DAKO Mounting Medium (DAKO, Denmark)

Visualization and image acquisition were performed on Leica DM6000 microscope with a DFC420 camera, and images were processed in Fiji package (Schindelin et al., 2012) based on ImageJ (NIH, United States).

### ELISA for Transforming Growth Factor β1

TGF-β1 concentration measurement was performed using Human/Mouse/Rat/Porcine/Canine TGF-β1 Quantikine ELISA Kit (DB100B, R&D Systems, United States) according to the manufacturer’s instructions. Samples containing 10% solution of MDS, BS, or FBS in DMEM/F12 (similar to solutions used for cell cultivation in our experiments) were centrifuged, and supernatants were stored at −80°Ñ. Immediately before use, samples have been activated to transform latent TGF-β1 to the immunoreactive form. For this purpose, 20 μl of 1 M HCl solution was added to 90 μl of each sample, and then it was neutralized by addition of 20 μl of 1.2 N NaOH, 0.5 M HEPES. The final pH was checked in obtained activated samples, so that it was within the pH 7.2–7.6 range. Then, some of these activated samples were additionally diluted for two or five times with appropriate calibration diluent, if needed. Further steps were carried out using the manufacturer’s instructions for the kit. For each measurement procedure, the calibration curve was built using TGF-β1 standard solutions. Each sample and standard measurement was performed in duplicate. The dilutions as a result of sample activation procedure (1.45-fold) and further two or fivefold dilutions were taken into consideration in the final calculation of TGF-β1 concentration in the samples.

### *In vitro* Model of Transforming Growth Factor β1-Induced Fibrogenesis

SCs were grown in a 24-well plate to 100% confluence, and medium was changed to one of the following or a corresponding control:

• DMEM/F12 (Gibco, United States) + 10 ng/ml TGF-β1 (8915, Cell Signaling Technology, United States) + 100 μM magnesium ascorbil phosphate (MAP, Sigma, United States) and control DMEM/F12 + 100 μM MAP were used to assess the fibrogenic effect of TGF-β1. MAP was added to serum-free medium as a coenzyme required for collagen I deposition (Chen et al., 2009).
• 10% BS in DMEM/F12 or 10% MDS in DMEM/F12 was used as tested media.
• 10% FBS (HyClone, United States) in DMEM/F12 was used as a control media to assess the effects of MDS and BS in a model of fibrogenesis.
• BS was used as negative control for soluble factors derived from the endometrium and contained in MDS.

The medium was changed every 48 h to maintain the concentration of its active components. At 96 h of the experiment, cells were either lysed by Laemmli buffer (BioRad, United States) to analyze collagen I and ED-A fibronectin content by Western blotting (see *Electrophoresis and Western Blotting*) or fixed with 4% formaldehyde (Panreac, United States) for immunofluorescent labeling of extracellular collagen I and ED-A fibronectin (see section “Immunofluorescent Labeling”).

### Electrophoresis and Western Blotting

Electrophoresis and transfer were performed using devices and reagents from BioRad. Electrophoresis was performed according to a method described by Laemmli (1970). Stacking (4% acrylamide) and resolving (12.5% acrylamide) gels were used with the ratio of acrylamide and methylene-bis-acrylamide in gels of 37.5:1.

After electrophoresis, proteins were transferred to a polyvinylidene fluoride (PVDF) membrane (Amersham, United States). Western blotting was performed according to the method described by Towbin et al. (1979). Electrotransfer of proteins to a nitrocellulose membrane was carried out for 16 h at a constant voltage of 30 V in Bjerrum and Schafer–Nielsen transfer buffer (48 mM Tris-HCl buffer, pH 9.2, containing 39 mM glycine and 20% ethanol) (Bjerrum, 1986). After transfer, membranes were blocked by 5% non-fat milk solution in PBST and incubated with primary antibody solutions to α-SMA (904601, Biolegend, United States), vinculin (V4139, Sigma, United States), glyceraldehyde 3-phosphate dehydrogenase (GAPDH; 2118s, Cell Signaling Technology, United States), SMAD2 (3122S, Cell Signaling Technology, United States), phospho-SMAD2 (3108S, Cell Signaling Technology, United States), collagen I (ab34710, Abcam, United Kingdom), or ED-A fibronectin (ab6328, Abcam, United Kingdom), followed by corresponding secondary antibody solutions (P-GAM Iss or P-GAR Iss, IMTEK, Russia). Detection was performed using Clarity ECL Solution (1705061, BioRad, United States) or ClarityMax ECL Solution (1705062, BioRad, United States) followed by visualization on ChemiDoc Touch (BioRad, United States). ImageLab software (BioRad, United States) was used for densitometry of the obtained results. Densitometry data were log-transformed (log_2_) after normalization to loading control and corresponding control sample.

### Immunofluorescent Labeling

Immunofluorescent labeling was performed directly in wells of culture plates. For confocal microscopy, we used special dishes with thin plastic bottoms (81156, Ibidi, Germany). Cells were fixed with 4% formaldehyde (Panreac, United States) during 10 min, then permeabilized for 10 min by 0.02% Triton-X100 (Panreac, United States) for α-SMA and vinculin labeling. For collagen I and ED-A fibronectin labeling, permeabilization stage was omitted. Blocking was done with 10% goat serum (Thermo Fisher Scientific, United States) solution in HBSS (Paneco, Russia) during 1 h. Overnight incubation with the first antibodies to α-SMA (ab5694, Abcam, United States), vinculin (V9264, Sigma, United States), collagen I (ab34710, Abcam, United Kingdom), or ED-A fibronectin (ab6328, Abcam, United Kingdom) was performed at 4°C. Incubations with the corresponding secondary antibodies (A21207 and A11001, Invitrogen, United States) lasted 2 h at room temperature. The nuclei were labeled with 4′,6-diamidino-2-phenylindole (DAPI; D9542, Sigma-Aldrich, United States). After each step, except for blocking, the wells were washed three times with phosphate buffer saline (PBS). Upon completion of labeling, PBS remained in the wells and samples were analyzed by microscopy.

Visualization and image acquisition were performed on a Leica DMi8 inverted fluorescence microscope with a DFC7000T camera. Confocal images were obtained on a Leica TCS SP5 confocal microscope, and water was used as an immersion medium. Image processing and analysis were completed using Fiji. Coloc2 plugin was used for assessing signal colocalization, 3D viewer plugin for 3D reconstruction, and 3D Object counter plugin for the evaluation of matrix protein levels of localization.

### Collagen Disc Contraction Assay

SCs were detached from the culture plastic as described above and centrifuged for 5 min at 200 g. The supernatant was discarded, cells were resuspended in PBS and counted, and the required number of cells (40 × 10^3^, 80 × 10^3^, or 160 × 10^3^) was placed in a 1.5 ml tube followed by centrifugation for 5 min at 200 g. The supernatant was discarded, the cells were resuspended in 400 μl of DMEM low glucose + 10 ng/ml TGF-β1 (8915, Cell Signaling Technology, United States) or DMEM low glucose (Gibco, United States) as corresponding control, then 200 μl of pork collagen (3 mg/ml in 0.1% acetic acid, Imtek, Russia) was added. Obtained suspensions were normalized with 1 M NaOH (Panreac, United States) and transferred into wells of a 24-well plate (500 μl in each well). The collagen in wells polymerized at room temperature for 30 min and then 400 μl of DMEM low glucose were added atop. After that, collagen discs were detached with a pipette tip from the walls of the wells to induce flotation and let contraction. Disc flotation was visually checked every 24 h, and the disc area was measured in photographs taken at 24, 48, 72, and 96 h after detachment. To assess collagen disc contraction, we calculated the percentage of collagen disc area relative to the plate well area (collagen disc area/well area × 100%). We designated the resulting value as “disc size.” At each time point, for each donor, the mean size of the discs with TGF-β1 and without TGF-β1 was calculated to plot the dynamics of mean disc size. Mean disc size for samples treated by TGF-β1 was estimated in each time point and compared vs. the corresponding control with evaluation of statistical significance for each type of SC.

### Statistical Analysis of Data

Data were analyzed in StatPlus v.7.3.3.0 (AnalystSoft Inc., United States). Kolmogorov–Smirnov test was used to evaluate normality of data distribution. *T*-test or Wilcoxon signed rank test for paired samples and *t*-test or Mann–Whitney test for independent samples was used for comparison of two samples. One-way ANOVA for comparison of >2 samples and Newman–Keuls test for multiple comparisons were used. Level of significance <0.05 was applied for all statistical comparisons.

## Results

### Endometrial Stromal Cells Undergo Transition to Myofibroblasts After Treatment With Transforming Growth Factor β1

To assess the ability of EndoSCs for myofibroblast transition, we used TGF-β1 as inducer and compared them vs. SCs from human dermis (DermSC) and subcutaneous fat (AdipoSC) in culture models of fibrogenesis and contraction. Prior to this comparative analysis, we investigated the compliance of isolated cells to previously described criteria of multipotent mesenchymal stromal cells (MSCs) (Dominici et al., 2006) to support direct comparisons of SCs from different tissues.

#### Stromal Cells From Human Endometrium, Dermis, or Adipose Tissue Share Mesenchymal Stromal Cell Immunophenotype and Demonstrate Multipotency *in vitro*

**FIGURE 1:**
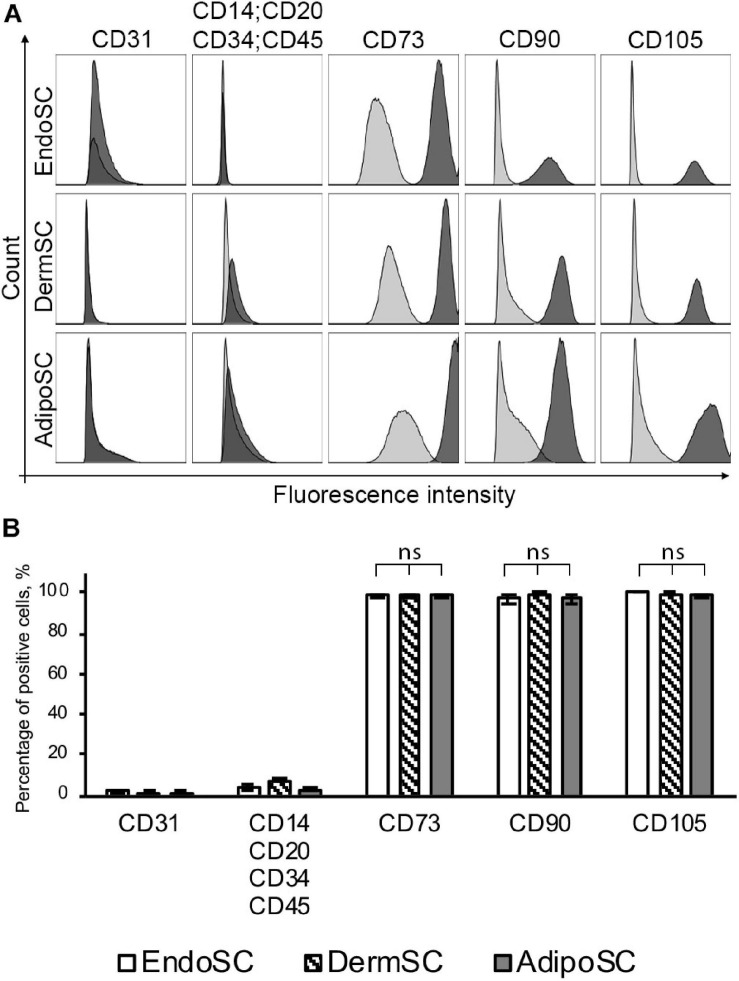
Stromal cells from the endometrium (EndoSCs), dermis (DermSCs), and adipose tissue (AdipoSCs) demonstrate similar immunophenotypes. **(A)** Representative histograms after flow cytometry of EndoSC, DermSC, and AdipoSC labeled for positive and negative markers of MSC. Distribution of signal intensity from specific antibodies (dark gray) or isotype control IgG (light gray) is plotted on histograms. **(B)** Quantitative analysis of phenotypic marker distribution in EndoSC, DermSC, and AdipoSC (*n* = 3 for each cell type). Data presented as mean ± SD; one-way ANOVA used; ns, non-significant statistical difference (*p* > 0.05).

At passages 3–5, all isolated SCs (*n* = 3 for each source) were positive for MSC markers (CD73, CD90, CD105) and negative for endothelial, immune, and hematopoietic cell markers (CD31, CD34, CD14, CD20, CD45) (Figure 1A). We found no significant difference between EndoSC, AdipoSC, and DermSC cultures with 95–100% cells displaying MSC immunophenotype (Figure 1B).

**FIGURE 2:**
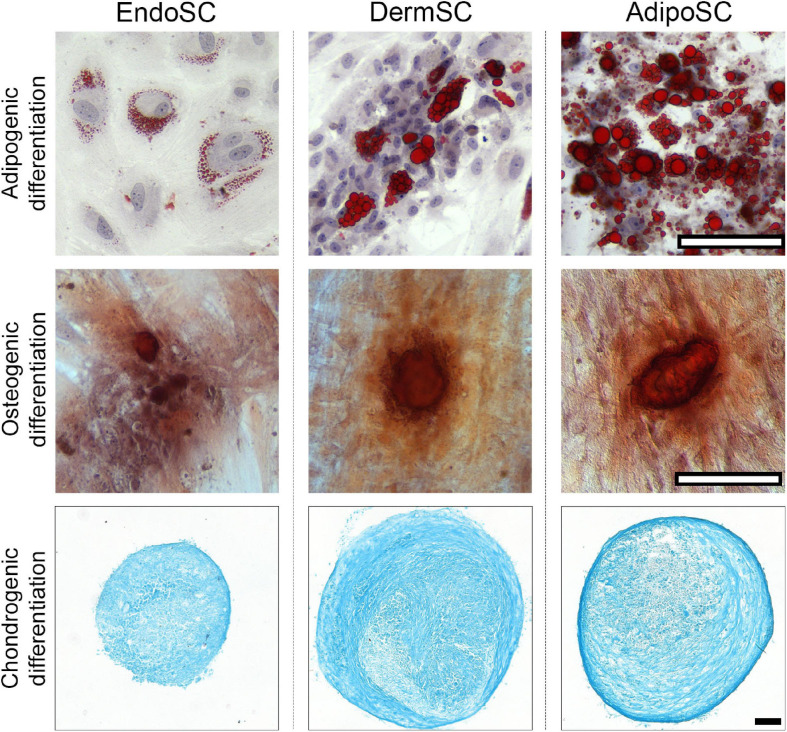
Stromal cells from the endometrium (EndoSCs), dermis (DermSCs), and adipose tissue (AdipoSCs) undergo adipogenic, osteogenic, and chondrogenic differentiation *in vitro*. Representative microphotographs of EndoSC, DermSC, and AdipoSC stained for lipid accumulation (Oil Red O) at day 14 of adipogenic differentiation, mineral depositions (Alizarin Red S) at day 21 of osteogenic differentiation, glycosaminoglycans (Alcian blue) at day 21 of chondrogenic differentiation; scale bar 100 μm.

After induction of adipogenic, chondrogenic, or osteogenic differentiation at passages 3–5, we found that independently of tissue source, all cultures treated by specific differentiation media accumulated lipid droplets, glycosaminoglycans or showed mineral deposition at respective endpoints (Figure 2), which reflected multipotency of obtained cell cultures.

We conclude that EndoSCs, DermSCs, and AdipoSCs matched accepted criteria of MSC and could be compared in further experiments as belonging to a common cell type.

#### Endometrial Stromal Cells Respond to Transforming Growth Factor β1 With SMAD2 Phosphorylation and Demonstrate a Distinct Pattern of Extracellular Matrix Organization

EndoSC, DermSC, and AdipoSC were taken at passages 3–5 and grown to confluence prior to modeling of fibrogenesis. At Day 0 of the experiment, we changed the medium to DMEM/F12 with TGF-β1 (10 ng/ml) supplemented with MAP (100 mM) to support ECM deposition as previously described (Chen et al., 2009). After that, cells were cultured for 96 h with a single change of culture medium at the midpoint of experiment to maintain nutrient and TGF-β1 levels. At the endpoint, activation of TGF-β1 signaling was evaluated and characteristic markers of transition to myofibroblasts (α-SMA, vinculin, collagen I, or ED-A fibronectin) were assayed in all SC cultures.

**FIGURE 3:**
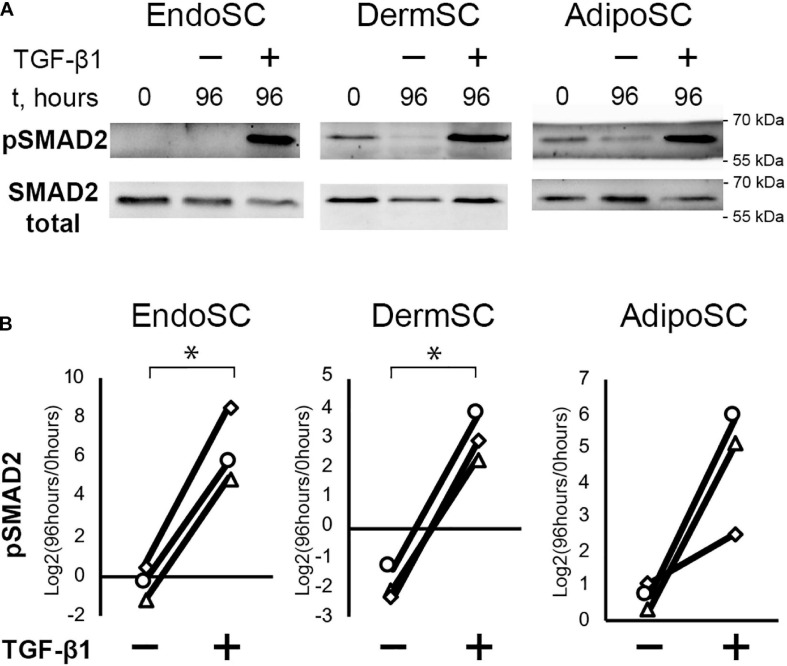
Treatment with transforming growth factor β1 (TGF-β1) induces SMAD2 signaling in stromal cells from the endometrium (EndoSCs), dermis (DermSCs), and adipose tissue (AdipoSCs). **(A)** Representative Western blotting of EndoSC, DermSC, and AdipoSC lysates obtained before TGF-β1 treatment (lane “0”), at 96 h in control culture (lane 96/–), or at 96 h after addition of 10 ng/ml TGF-β1 (lane 96/+). **(B)** Densitometry analysis of Western blotting for pSMAD2 in stromal cells demonstrating the effect of TGF-β1. Individual donor (*n* = 3) data presented as fold change relative to the level before TGF-β1 treatment (logarithmic scale) with SMAD2 loading control used for normalization. Paired *t*-test was used for statistical analysis of log-transformed data; ^∗^*p* < 0.05.

Treatment with TGF-β1 increased the level of phosphorylated SMAD2 (pSMAD2), indicating activation of canonical TGF-β1 signaling pathway in all cultures including EndoSC (Figures 3A,B; Kim et al., 2018). Thus, SCs from the endometrium showed a typical signaling response to TGF-β1 similarly to cells from other sources.

**FIGURE 4:**
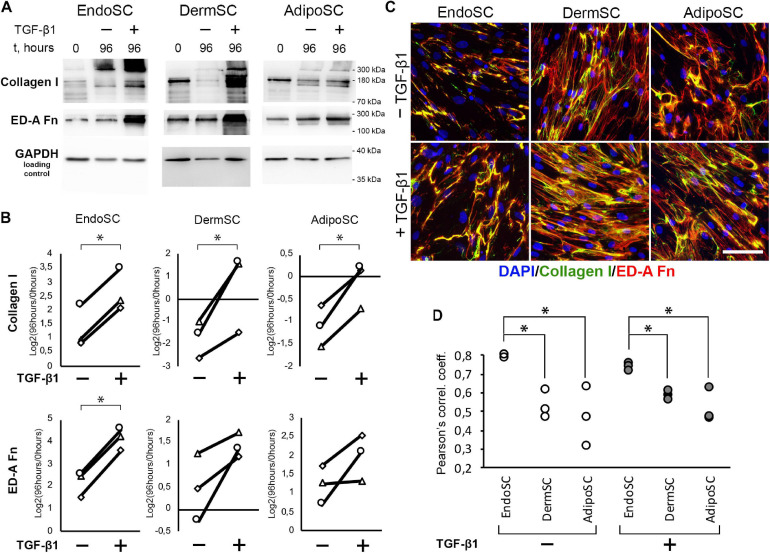
Endometrial stromal cells (EndoSCs) increase collagen I and ED-A fibronectin production in response to transforming growth factor β1 (TGF-β1) treatment with a distinct pattern of extracellular matrix (ECM) deposition. **(A)** Representative Western blotting of EndoSC, dermal stromal cell (DermSC), and adipose tissue stromal cell (AdipoSC) lysates obtained before TGF-β1 treatment (lane 0), at 96 h in control culture (lane 96/–), or at 96 h after addition of 10 ng/ml TGF-β1 (lane 96/ +). **(B)** Densitometry analysis of ECM components in stromal cells shown in panel **(A)** at experimental endpoint (96 h). Individual donor data (*n* = 3) presented as fold change relative to the level before TGF-β1 treatment (logarithmic scale) with glyceraldehyde 3-phosphate dehydrogenase (GAPDH) loading control used for normalization. Paired *t*-test was used for statistical analysis of log-transformed data; ^∗^*p* < 0.05. **(C)** Immunofluorescence of collagen I and ED-A fibronectin in EndoSC, DermSC, and AdipoSC cultures treated with TGF-β1 and untreated control. Scale bar 100 μm. **(D)** Higher level of collagen I and ED-A fibronectin colocalization in EndoSC than in DermSC and AdipoSC in control and TGF-β1-treated cultures; Pearson’s correlation coefficient for three repeats, one-way ANOVA and Newman–Keuls test were used for statistical analysis; ^∗^*p* < 0.05.

Besides TGF-β1 signaling measurement, we used immunoblotting to evaluate level changes of ED-A fibronectin and collagen I in SC cultures in response to TGF-β1 (Figures 4A,B). Statistical significance for ED-A fibronectin was reached only in EndoSC, which reflected minimal donor-to-donor variability of results. Similar tendency was observed and reproduced in all three repeats for DermSC and AdipoSC.

As far as immunoblotting of total culture lysates fails to discern between intracellular and extracellular ECM components, we addressed immunofluorescence studies. To visualize extracellular deposition of collagen I and ED-A, we omitted permeabilization stage during immunofluorescent labeling.

As seen in Figure 4C, each cell culture demonstrated pronounced deposition of collagen I and ED-A fibronectin. Visual assessment of micrographs suggested that deposition of collagen I and ED-A fibronectin increased after TGF-β1 treatment and EndoSC showed a response comparable to that of SCs from other tissues (Figure 4C).

In general, TGF-β1 increased production and deposition of ECM proteins in EndoSC as well as in DermSC and AdipoSC in a similar manner. However, the observed pattern of collagen I and ED-A fibronectin deposition was peculiar in EndoSC. Indeed, analysis of merged images (Figure 4C) suggested that only in EndoSC signal from collagen I and ED-A fibronectin was strongly colocalized in contrast with DermSC and AdipoSC. We supported this observation by additional correlative analysis of colocalization for collagen I and ED-A fibronectin and found that independently of TGF-β1 treatment, the correlation coefficient was significantly higher in EndoSC compared to that of DermSC or AdipoSC (Figure 4D). This provided grounds for assumption that autonomous tissue-specific properties of EndoSC define a distinct pattern of ECM organization resulting in highly colocalized deposition of collagen I and ED-A fibronectin.

#### Endometrial Stromal Cells Acquire Contractile Myofibroblast Phenotype in Response to Transforming Growth Factor β1

**FIGURE 5:**
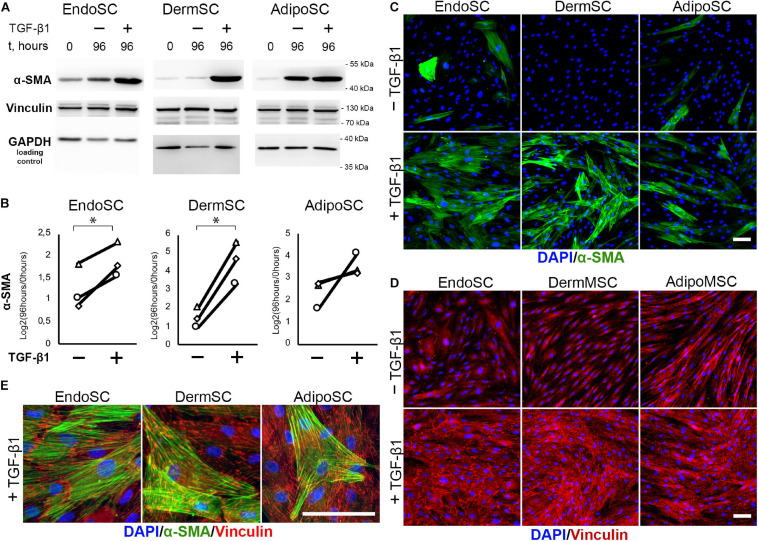
Stromal cells from the endometrium (EndoSCs), dermis (DermSCs), and adipose tissue (AdipoSCs) acquired myofibroblast phenotype after transforming growth factor β1 (TGF-β1) treatment. **(A)** Western blotting analysis of α-smooth muscle actin (α-SMA) and vinculin in EndoSC, DermSC, and AdipoSC lysates obtained before TGF-β1 (lane 0), at 96 h in control culture (lane 96/–), or at 96 h after addition of 10 ng/ml TGF-β1 (lane 96/+). **(B)** Densitometry analysis of α-SMA changes in stromal cells treated by TGF-β1 shown in panel **(A)**. Individual donor data (*n* = 3) presented as fold change relative to the level of the evaluated protein before TGF-β1 treatment (logarithmic scale) with glyceraldehyde 3-phosphate dehydrogenase (GAPDH) loading control used for normalization. Paired *t*-test was used for statistical analysis of log-transformed data; ^∗^*p* < 0.05. **(C)** Immunofluorescence of α-SMA in EndoSC, DermSC, and AdipoSC cultures treated with TGF-β1 and in untreated control. Scale bar 100 μm. **(D)** Immunofluorescence of vinculin in EndoSC, DermSC, and AdipoSC cultures treated with TGF-β1 and in untreated control. The same field of view as in panel **(C)** is presented. Scale bar 100 μm. **(E)** Merged high-magnification images after immunofluorescent labeling of α-SMA and vinculin in EndoSC, DermSC, and AdipoSC cultures treated by TGF-β1. Scale bar 100 μm.

Treatment with TGF-β1 resulted in an increase of α-SMA in all tested cultures including EndoSC, while increment of α-SMA level after exposure to TGF-β1 was maximal in DermSC (Figure 5A). Statistical significance was reached only for EndoSC and DermSC, while in AdipoSC, a reproducible trend was observed not reaching critical *p*-value of 0.05 (Figure 5B). Immunofluorescence showed that in all SC cultures treated with TGF-β1, α-SMA was prominently present in cellular stress fibers (compared to untreated control), and the biggest difference was detected in DermSC supporting Western blot results (Figure 5C). Overall, TGF-β1 increased the amount of α-SMA in stress fibers in EndoSC, DermSC, and AdipoSC which supported enhanced by ECM deposition demonstrated earlier (Figure 4) suggested acquisition of myofibroblast phenotype.

One more interesting observation was that the level of α-SMA spontaneously increased in all SC cultures during serum deprivation that preceded our experiment (Figure 5A; see difference between 0 and 96 h in untreated controls). In DermSC, this phenomenon was marginally absent in immunofluorescence and Western blotting, suggesting their tissue-specific resistance to spontaneous transformation to myofibroblasts under serum deprivation.

The level of vinculin assayed by Western blotting did not change after incubation with TGF-β1 (Figure 5A) but in SCs treated by TGF-β1 immunofluorescence detected enlargement of its clusters (Figures 5D,E) characteristic of focal adhesion contacts and transition of SCs to myofibroblasts (Hinz et al., 2019).

#### Endometrial Stromal Cells Demonstrate Contraction Ability Similar to That of Other Stromal Cells

Contractile function characteristic of myofibroblasts was assessed in a model that relies on size change in a floating disc consisting of porcine collagen with embedded EndoSC, DermSC, and AdipoSC for 96 h. Discs were supplemented with 10 ng/ml of TGF-β1 to induce contraction. To establish optimal cell density for contraction measurement, we titrated cells in the range of 160×10^3^–40×10^3^. Contraction was assessed by disc size change.

**FIGURE 6:**
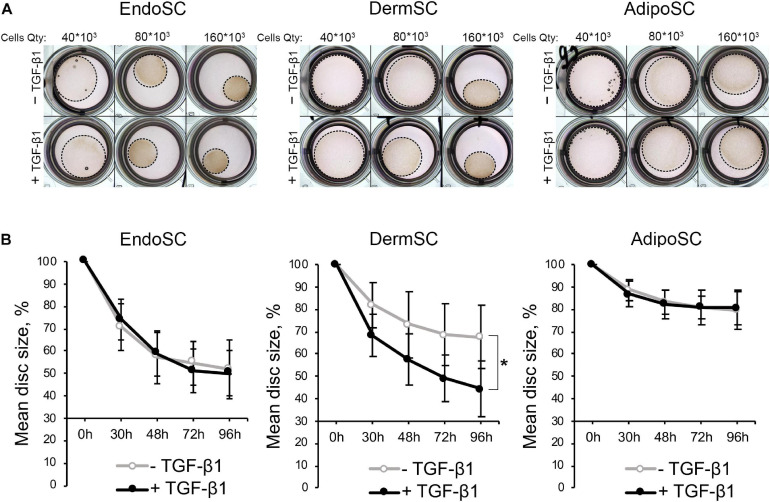
Contraction of collagen disc by control and transforming growth factor β1 (TGF-β1)-treated stromal cells from the endometrium (EndoSCs), dermis (DermSCs), or adipose tissue (AdipoSCs). **(A)** Representative photographs of porcine collagen discs embedded with stromal cells in different quantities (40 * 10^3^, 80 * 10^3^, 160 * 10^3^) taken at 48 h of experiment in TGF-β1-treated and control groups. **(B)** Graphs reflecting basal contraction of discs embedded with EndoSC, DermSC, or AdipoSC and effect of TGF-β1. Disc size (%) was calculated as the area of disc/area of well. Data presented as mean ± SD; paired *t*-test was used for statistical analysis; *p < 0.05.

Generally, all used SCs were able to contract collagen without TGF-β1 treatment, while the impact of TGF-β1 on disc contraction was tissue-specific. For EndoSC and AdipoSC, TGF-β1 did not influence contraction; however, contraction of DermSC-embedded discs was enhanced by TGF-β1 compared to control (Figures 6A,B). Interestingly, untreated EndoSC contracted collagen discs to marginally the same size as demonstrated by DermSC exposed to TGF-β1.

Observed changes of contraction in DermSC after addition of TGF-β1 were concordant with previous Western blotting and immunofluorescence results (Figure 5). Response of DermSC to TGF-β1 with pronounced increase of contractility may result from tissue-specific extremely low basal production of α-SMA until simulation by TGF-β1 (Figure 5). Absence of this feature in EndoSC and AdiposSC resulted in spontaneous contraction of discs without addition of TGF-β1. Overall, we concluded that all SCs including EndoSC possessed functional contractile ability under TGF-β1 treatment, which was accompanied by the appearance of myofibroblast ECM markers collagen I and ED-A fibronectin (Figure 5).

### Soluble Factors Produced During Menstruation May Interrupt Transition of Stromal Cells to Myofibroblasts

To understand the impact of soluble factors produced by the endometrium during menstruation, we used MDS that contained local factors produced *in utero* and systemic stimuli released from the blood flow. MDS influence was evaluated in a set of fibrogenesis models involving transition of SCs to myofibroblasts. To reveal specific effects of MDS reflecting the contribution of the environment *in utero*, we used venous BS as a negative control. BS and MDS samples were obtained from donors on the second day of menstruation.

#### Both Menstrual Discharge Serum and Venous Blood Serum Contain High Concentrations of Transforming Growth Factor β1

**FIGURE 7:**
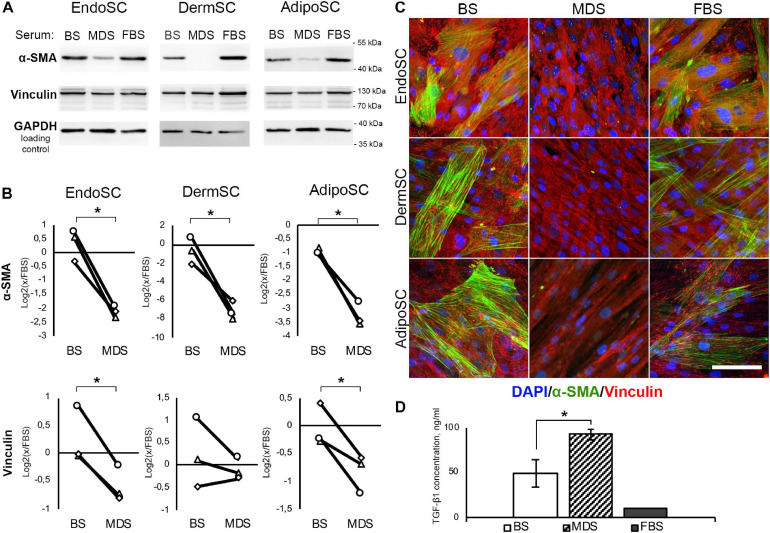
Local factors produced during menstruation inhibit phenotypic transition to myofibroblasts in stromal cells from the endometrium (EndoSCs), dermis (DermSCs), or adipose tissue (AdipoSCs). **(A)** Representative Western blotting of α-smooth muscle actin (α-SMA) and vinculin in lysates of EndoSC, DermSC, and AdipoSC treated with 10% venous blood serum (BS), menstrual discharge serum (MDS), or fetal bovine serum (FBS) at 96 h of experiment. **(B)** Densitometry of α-SMA and vinculin changes in stromal cells treated by serum preparations shown in panel **(A)**. Individual donor data (*n* = 3) presented as fold change relative to the evaluated protein level in control sample treated with FBS (logarithmic scale); glyceraldehyde 3-phosphate dehydrogenase (GAPDH) loading control used for normalization. Paired *t*-test was used for statistical analysis of log-transformed data; *p < 0.05. **(C)** Immunofluorescence of α-SMA and vinculin in EndoSC, DermSC, and AdipoSC treated with 10% BS, 10% MDS, or 10%FBS; 96 h of experiment. Scale bar 100 μm. **(D)** Concentrations of transforming growth factor β1 (TGF-β1) in MDS, BS, and FBS measured by ELISA. Mann–Whitney test was used for statistical analysis; *p < 0.05.

Prior to application of MDS or BS in relevant models, we have assayed obtained preparations for contents of TGF-β1 by ELISA. Used in further experiments, 10% solution of MDS in DMEM/F12 contained 9.3 ± 0.57 ng/ml of TGF-β1, which was similar to 10 ng/ml used in our models described above and was actually sufficient to induce transition to myofibroblasts. Moreover, TGF-β1 concentration in 10% solution of BS in DMEM/F12 used in the same experiments was nearly twofold lower than in 10% MDS and was equal to 4.9 ± 0.15 ng/ml (Figure 7D).

#### Menstrual Discharge Serum Prevents Acquisition of Contractile Phenotype by Stromal Cells Derived From Endometrium, Dermis, and Adipose Tissue

The abundance of TGF-β1 in both MDS and BS samples (Figure 7D) along with demonstrated sensitivity of all tested cells to this growth factor (Figures 3, 5, 6) allowed us to test these preparations without supplementation of TGF-β1 on cell cultures.

In all SCs, treatment with MDS leads to a decrease of α-SMA (immunoblotting) and the reduction of α-SMA-positive stress fibers (immunofluorescence) compared to BS (Figures 7A–C). Vinculin clusters also became less prominent after MDS treatment, which correlated with the assumption that it may interrupt acquisition of a myofibroblast phenotype (Figure 7C). After incubation with 10% MDS, the vinculin content in EndoSC and AdipoSC slightly decreased (Figures 7A,B). This represents a notable finding, assuming the major role of vinculin in myofibroblast function.

Altogether, we suggest that soluble factors within MDS counteract transition of SCs to contractile myofibroblasts in response to profibrotic factors.

#### Treatment of Stromal Cells by Menstrual Discharge Serum Decreases Collagen I Production and Facilitates Its Extracellular Deposition

**FIGURE 8:**
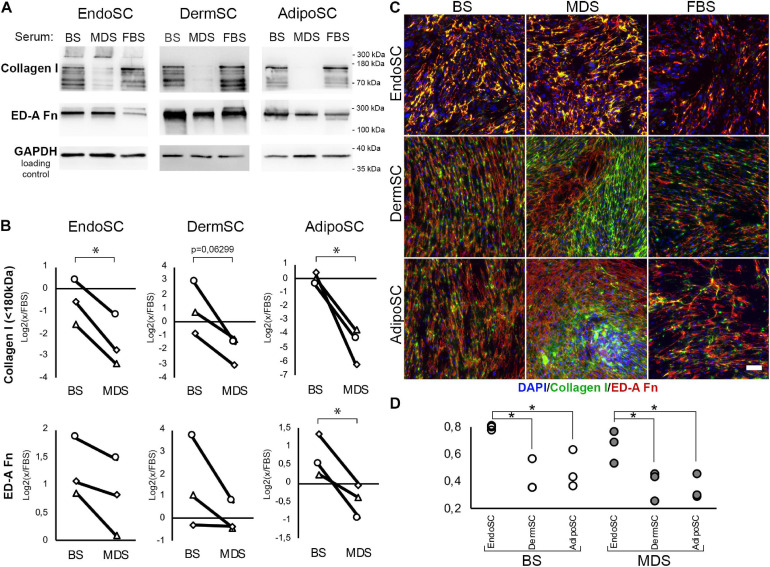
Extracellular matrix (ECM) deposition and its tissue-specific patterns after treatment of stromal cells by menstrual blood discharge or peripheral venous blood serum. **(A)** Representative Western blotting of endometrial stromal cell (EndoSC), dermis stromal cell (DermSC), and adipose tissue stromal cell (AdipoSC) lysates treated with 10% blood serum (BS), menstrual discharge serum (MDS), or fetal bovine serum (FBS) at 96 h of experiment. **(B)** Densitometry of extracellular matrix (ECM) components in stromal cells treated by serum preparations shown in panel **(A)**. Individual donor data (*n* = 3) presented as fold change relative to the evaluated protein level in control sample treated with FBS (logarithmic scale); glyceraldehyde 3-phosphate dehydrogenase (GAPDH) loading control used for normalization. Paired *t*-test was used for analysis of log-transformed data; *p < 0.05. **(C)** MDS induces anisotropic deposition of extracellular collagen I in stromal cells from the dermis and adipose tissue, but not endometrium. In contrast, stromal cells cultured with BS demonstrated a homogeneous deposition of collagen I and ED-A fibronectin. For EndoSC, response to MDS or BS demonstrates minimal differences and vividly homogeneous deposition of ECM after both treatments. Merged extracellular collagen I and ED-A fibronectin immunofluorescence; nuclei stained with 4′,6-diamidino-2-phenylindole (DAPI). Scale bar 100 μm. **(D)** Immunfluorescence of extracellular collagen I and ED-A fibronectin demonstrated a higher level of colocalization in EndoSC compared to DermSC and AdipoSC independently of serum treatment used in cultures; Pearson’s correlation coefficient for three repeats; one-way ANOVA and Newman–Keuls test were used for statistical analysis; *p < 0.05.

To assess the influence of local factors produced during menstruation on ECM production by SCs, we evaluated collagen I and ED-A fibronectin after treatment with MDS or BS. Cells were cultured for 96 h in DMEM/F12 supplemented by 10% of either MDS or BS. Treatment with 10% FBS was used as the baseline culture condition control. Western blotting demonstrated that in all SC cultures, MDS decreased the amount of non-aggregated, full-length (approximately 130 kDa) type I collagen molecules compared to BS (Figure 8A). Statistical significance was reached for EndoSC and AdipoSC, while in DermSC, a reproducible tendency was observed with a marginal level of significance (Figure 8B). A reproducible concordant decrease of ED-A fibronectin was found by immunoblotting in all MDS-treated cultures, yet statistical significance in comparison to BS samples was reached only for AdipoSC.

Immunofluorescent labeling without permeabilization demonstrated enhanced extracellular deposition of collagen I and ED-A fibronectin in all studied SCs after treatment with either MDS or BS (Figure 8C). This does not contradict our immunoblotting data on the reduction of non-aggregated, full-length (approximately 130 kDa) collagen I in total culture lysates (Figures 8A,B) because Western blotting detects total collagen I amount in culture and not only its deposition in ECM synthesized by SCs. High-molecular-weight type I collagen aggregates (approximately 300 kDa band) attributed to extracellular large-scale complexes were detected exclusively in EndoSC lysates (Figure 8A). Treatment with MDS resulted in enhanced signal in this band, which corresponds to intensive immunofluorescent signal from collagen I in MDS-treated EndoSC (Supplementary Figure 1).

Finally, we found that MDS, but not BS, induced anisotropic deposition of collagen I (Figure 8C) with uneven distribution of signal, indicating high and low density of its incorporation in ECM. Areas of high cell density with increased collagen I deposition were formed only in AdipoSC and DermSC and exclusively after treatment with MDS, while FBS or BS failed to induce the described changes. Under MDS influence, EndoSC showed enhanced homogeneous deposition of ECM with a persistent pattern characterized by a high degree of colocalization of collagen I and ED-A fibronectin, independently of serum used (Figures 8C,D). This reproduces our data from experiments with TGF-β1 alone (Figure 4D) suggesting that high colocalization of collagen I and ED-A fibronectin is an intrinsic property of EndoSC exerted independently of stimulus, while other SCs respond differentially to MDS or BS.

### Tissue-Specific Response of Stromal Cells to Menstrual Discharge Serum Involves Extracellular Matrix Spatial Distribution and Formation of “Hill” Morphology

Analysis of merged images after labeling for collagen I and ED-A fibronectin showed anisotropic deposition of collagen I in DermSC and AdipoSC treated by MDS, but not BS. In areas with high cellular density, the fibrillar structure of collagen I was enhanced, while in EndoSC cultures, we did not observe this feature (Supplementary Figure 2). Abovementioned high-density areas possessed a characteristic morphology for structures historically termed as “hills.” Briefly, “hills” are multilayered entities formed in stromal or smooth muscle cell cultures during relatively long periods in culture (12–14 days) without passaging with regular changes of FBS-supplemented medium. Notably, in AdipoSC and DermSC cultures with medium that contained 10% MDS, but not 10% BS, we observed a rapid formation of “hills” (Figure 8C). It occurred as early as day 4 compared to approximately 14 days typically observed in FBS-supplemented media (Nimiritsky et al., 2019).

**FIGURE 9:**
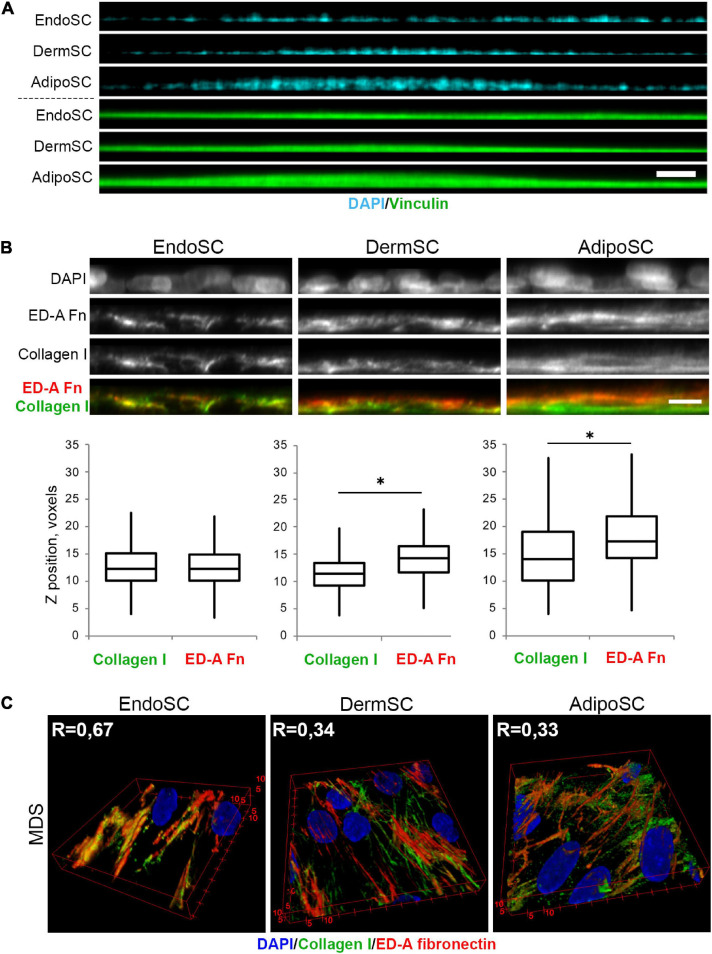
Tissue-specific features of extracellular matrix (ECM) spatial organization in stromal cells treated by menstrual discharge serum (MDS). **(A)** Treatment with MDS induces formation of “hills” in dermis stromal cells (DermSCs) and adipose tissue stromal cells (AdipoSCs), but not in endometrium stromal cell (EndoSC) cultures. Vinculin was used as a background labeling antigen to visualize stromal cells; nuclei stained by 4′,6-diamidino-2-phenylindole (DAPI). Confocal microscopy with side projection of resliced z-stacks. Scale bar 50 μm. **(B)** After MDS treatment, spatial organization of ECM is characterized by superficial localization of ED-A fibronectin above collagen I in DermSCs and AdipoSCs, while EndoSCs lack this feature, displaying homogeneous deposition of these ECM components. Immunofluorescent staining without permeabilization. Side projection of resliced z-stacks. Scale bar 10 μm; confocal microscopy. Whisker-box plots present the distribution of collagen I and ED-A fibronectin localization. Wilcoxon signed rank test was used for statistical analysis; ^∗^*p* < 0.05. **(C)** Higher level of collagen I and ED-A fibronectin colocalization (Pearson’s correlation coefficient, R) in EndoSC than in DermSC and AdipoSC in MDS-treated cultures. 3D reconstruction of collagen I and ED-A fibronectin deposition pattern. Confocal microscopy.

Taking that into account, we used MDS-treated SCs to assess their tissue-specific features that might be enhanced by regulatory stimuli within this fluid preparation. Using confocal microscopy, we found that in “hills” formed by MDS-treated AdipoSC and DermSC, collagen I was predominantly localized within the bottom layers while ED-A fibronectin was deposited close to the surface. In contrast, we observed that in EndoSC, which never displayed “hill-like” formations (Figure 9A), these ECM components showed no spatial specificity and were localized at the same *Z*-axis level (Figure 9B). These sets of data allowed to perform a 3D reconstruction of collagen I and ED-A fibronectin deposition that confirmed a high level of their colocalization in MDS-treated EndoSC, but not in DermSC or AdipoSC after the same treatment (Figure 9C).

Thus, the previously observed features of ECM organization by EndoSC and their inability to form “hills” were accompanied by a specific morphogenic response to soluble factors that were isolated along with MDS. These distinct differences of EndoSC from AdipoSC and DermSC were especially prominent after MDS treatment. However, we also found that effects of MDS on collagen I and ED-A fibronectin production lack cell specificity, indicating that all used SC cultures show concordant changes induced by soluble factors of MDS (Figure 8). At the same time, in AdipoSC and DermSC, “hill” formation was accelerated by MDS, while EndoSC still lacked this feature under all serum samples including control FBS.

## Discussion

In the present work, we investigated physiological factors that may determine the absence of fibrosis during endometrium healing. According to our data, the absence of damage-induced fibrosis in the endometrium can hardly be attributed to a resistance of EndoSCs to TGF-β1, which is a well-characterized profibrotic growth factor. In our experiments, TGF-β1 activated the SMAD2 pathway in all studied SCs, including EndoSC, indicating their sensitivity at the reception and signaling levels. TGF-β1 induced transition to myofibroblasts in SCs from the endometrium, dermis, and adipose tissue with a typical phenotype.

For each type of SCs used in our work, the ability to acquire myofibroblast phenotype has been previously shown (Montesano and Orci, 1988; Nasu et al., 2005; Kakudo et al., 2012), but to our knowledge, our study is the first to directly compare the changes in phenotype, matrix deposition, and contractility in these SCs. We found that EndoSC and AdipoSC, in contrast to DermSC, increased α-SMA level during serum deprivation. This tissue-specific feature of DermSC explained the TGF-β1-induced increase of contraction observed only for this SC type in the contraction assay where serum deprivation was used as a condition for negative controls.

Having shown the sensitivity of EndoSC to TGF-β1 and their comparable to other SCs ability to acquire myofibroblast phenotype, we proceeded to study the possible contribution of soluble regulatory factors affecting the course of endometrial healing using MDS, which inevitably contained (at least a portion of) these soluble factors. First of all, we evaluated TGF-β1 content in the obtained serum samples and found that MDS contained about 95 ng/ml TGF-β1, which is enough to induce transformation of SCs into myofibroblasts according to *in vitro* protocols typically requiring not more than 10 ng/ml. Interestingly, peripheral BS contained merely half of TGF-β1 contents in MDS. Probably, production of this growth factor by cells of the damaged uterine wall has a dramatic contribution to its concentration in MDS (Omwandho et al., 2010).

Taking into account the vivid ability of EndoSC to myofibroblast differentiation in response to TGF-β1, we assumed that *in utero* EndoSC might be exposed to regulatory stimuli that prevent this process and ensure healing of the endometrium without fibrosis, unlike other soft tissues.

Our data about effects of MDS on SCs confirmed our assumption about the presence of soluble factor(s) that may suppress transition to myofibroblasts. Together with the results mentioned above, this suggests that soluble MDS components are indispensable for endometrial scar-free healing. Otherwise, the latter outcome would be unlikely given the high TGF-β1 content we found in MDS and pronounced mechanical stress due to contractions of uterine smooth muscles during menstruation and after labor (Maybin and Critchley, 2015).

Despite the fact that the nature of regulatory stimuli that determine the antifibrotic effect of MDS in SCs remains highly enigmatic, we can assume that their main targets are downregulation or modulation of mechanical stimulus reception (mechanosensing) and response to ones. In our experiments, we found that under the influence of MDS containing soluble factors secreted during menstruation, the formation of focal adhesion contacts, as well as levels of α-SMA and vinculin, is suppressed. In SCs, mentioned molecular targets comprise a system necessary for mechanosensing, mediating resistance to mechanical forces and subsequent transformation into myofibroblasts (Hinz et al., 2019). Decreased collagen I production and its more pronounced organization in the extracellular space under the influence of MDS support our assumption about suppressive effects of its components on the transition of SCs to myofibroblasts. Excessive production of matrix proteins by myofibroblasts (including collagens and fibronectin) makes a key contribution to fibrosis; in addition, they create the basis for granulation tissue and its subsequent remodeling into the scar during wound healing (Midwood et al., 2004; Hinz, 2016b).

Soluble factors formed during menstruation suppressed the transition to myofibroblasts not only in endometrial SCs. This phenomenon was also observed when MDS was added to cultures of SCs isolated from fat and dermis, which excludes tissue-specific suppressive action of MDS components on myofibroblast transition. Once putative antifibrotic factors in MDS are identified, this will provide ground for a hypothetical possibility of their application to suppress fibrogenesis not only in the uterus but also in other organs and tissues.

Suppression of new myofibroblasts formation can lead to decreased fibrosis or scarring. However, to end up with regeneration, healing must involve morphogenetic processes in the damaged area to induce replacement of functional structures (Owlarn et al., 2017). SCs are actively involved in morphogenesis by activating the growth of nerves and blood vessels, interacting with stem and immune cells, and by secreting growth factors and cytokines (Sagaradze et al., 2019; Makarevich et al., 2020) that form gradients of chemotactic and navigation stimuli.

Production of matrix proteins by SCs is one of their main morphogenetic features that is necessary for spatial organization of cells in the forming tissue (Nimiritsky et al., 2019). Our comparative analysis revealed significant differences in patterns of matrix organization between EndoSC and AdipoSC or DermSC. In particular, the ECM produced by EndoSC was characterized by a stronger degree of colocalization of collagen I and ED-A fibronectin than in the AdipoSC and DermSC.

Specific features of ECM composition and organization by SCs regulate their functional state through feedback mediated by integrin signaling and form a unique topography of connective tissue and determine its interaction with other cell types (Mouw et al., 2014). Thus, properties of matrix produced by EndoSC can affect not only fibrogenesis but also angiogenesis and epithelialization, which are important for endometrial healing and provide an intriguing task for investigation of mechanisms in this field (Fan et al., 2008; Owusu-Akyaw et al., 2019; Yin et al., 2019; Ghosh et al., 2020).

Another morphogenetic feature of EndoSC is their inability to form “hills,” which are, actually, bulky connective tissue structures. Formation of such structures occurs during healing in the early stages during granulation tissue growth (Midwood et al., 2004). In most human organs, with the exception of bones, formation of granulation tissue leads to its remodeling and scar formation in the later stages. Inability of EndoSC to form “hills” might be a reflection of tissue-specific properties of this cell type, namely, reduced connective tissue growth. This corresponds with the absence of granulation tissue described during healing of damaged endometrium and, putatively, absence of its scarring in this tissue. At the same time, it is known that in animals capable of epimorphic regeneration, damage of soft tissues induces growth of voluminous connective tissue structures. Formation of bulky connective structures occurs at early stages of limb regeneration in axolotl and during heart regeneration in *Danio rerio* (González-Rosa et al., 2017; Haas and Whited, 2017). However, in these species, after formation and growth of connective tissue, scarring does not occur and healing goes into successful regeneration due to migration, dedifferentiation, redifferentiation, and reorganization of cells in a process reminiscent of embryonic morphogenesis.

Tissue-specific differences in the morphogenetic properties of SCs described above became more pronounced under the influence of MDS and its soluble factors. Indeed, MDS significantly accelerated the formation of “hills” in AdipoSC and DermSC but did not induce their appearance in EndoSC cultures; in contrast, soluble factors of peripheral BS did not show this effect. In addition, MDS induced the formation of a prominent fibrillar network of collagen I in the “hills,” which suggested a higher degree of matrix maturity, which is a characteristic of formed connective tissues (Mouw et al., 2014).

Thus, soluble factors formed during menstruation induce a tissue-specific morphogenic program in different SCs. Our data on cellular models may leave space for dispute on how this effect is realized *in vivo*, but intriguing assumption could be made: possibly, different tissue-specific regenerative programs are regulated and induced by similar regulatory stimuli. Evans et al. (2019) has shown that MDS can stimulate epithelialization of injured skin. Our results support the need to study the impact of MDS on outcomes of injury response in such animal models where healing normally ends by scarring. Obviously, if MDS contains soluble factors able to induce scar-free healing, these factors should be identified in future works. In addition, more study is required to understand what determines morphogenetic properties of EndoSC associated with the formation of bulk connective tissue structures and to determine if they are important or not for resistance of the endometrium to growth of granulation tissue and scarring.

## Conclusion

From the point of view of the physiology of the healing process of the endometrium, we can assume that the factors contained in the menstrual discharge prevent transition of SCs into myofibroblasts and activate their tissue-specific morphogenetic program. These two components are likely to ensure the rapid regeneration of this tissue and its subsequent preparation for the realization of reproductive function.

## Data Availability Statement

The raw data supporting the conclusions of this article will be made available by the authors, without undue reservation.

## Ethics Statement

The studies involving human participants were reviewed and approved by the Ethics Committee of Lomonosov Moscow State University. The patients/participants provided their written informed consent to participate in this study.

## Author Contributions

VT and PM: study supervision and funding acquisition. RE study concept. PM and RE: study design and wrote the manuscript. RE: microscopy, immunofluorescence, MDS, BS, and EndoSC isolation, cell culture, fibrogenesis assay, data analysis. MK: electrophoresis and western blotting. NA and OG: isolation of DermSC and AdipoSC. NA: cell culture. PN and ME: protein assays and ELISA. NB: collagen disc contraction assay. OG: cell differentiation. DD: flow cytometry. All authors contributed for the development of this article.

## Conflict of Interest

The authors declare that the research was conducted in the absence of any commercial or financial relationships that could be construed as a potential conflict of interest.
