# Supplementary material for: Scar-Free Healing of Endometrium: Tissue-Specific Program of Stromal Cells and Its Induction by Soluble Factors Produced After Damage
Source: Front Cell Dev Biol. 2021 Feb 25;9:616893. doi: 10.3389/fcell.2021.616893 (PMC7947248; doi:10.3389/fcell.2021.616893)
Supplement: Supplementary file 1 [file Data_Sheet_1.docx]

Supplementary Material

**Supplementary figure 1**. Stronger signal from collagen I in EndoSC than in DermSC and AdipoSC. Extracellular collagen I immunofluorescence; nuclei stained with DAPI. Scale bar 100 μm.

**Supplementary figure 2.** Enhanced visualization of collagen I fibrillar structure after MDS, but not BS treatment in DermSC and AdipoSC, but not EndoSC cultures. Merged extracellular collagen I and ED-A fibronectin immunofluorescence; nuclei stained with DAPI. Scale bar 100 μm.
