# Supplementary figures and images for: Scar-Free Healing of Endometrium: Tissue-Specific Program of Stromal Cells and Its Induction by Soluble Factors Produced After Damage
Source: Front Cell Dev Biol. 2021 Feb 25;9:616893. doi: 10.3389/fcell.2021.616893 (PMC7947248; doi:10.3389/fcell.2021.616893)

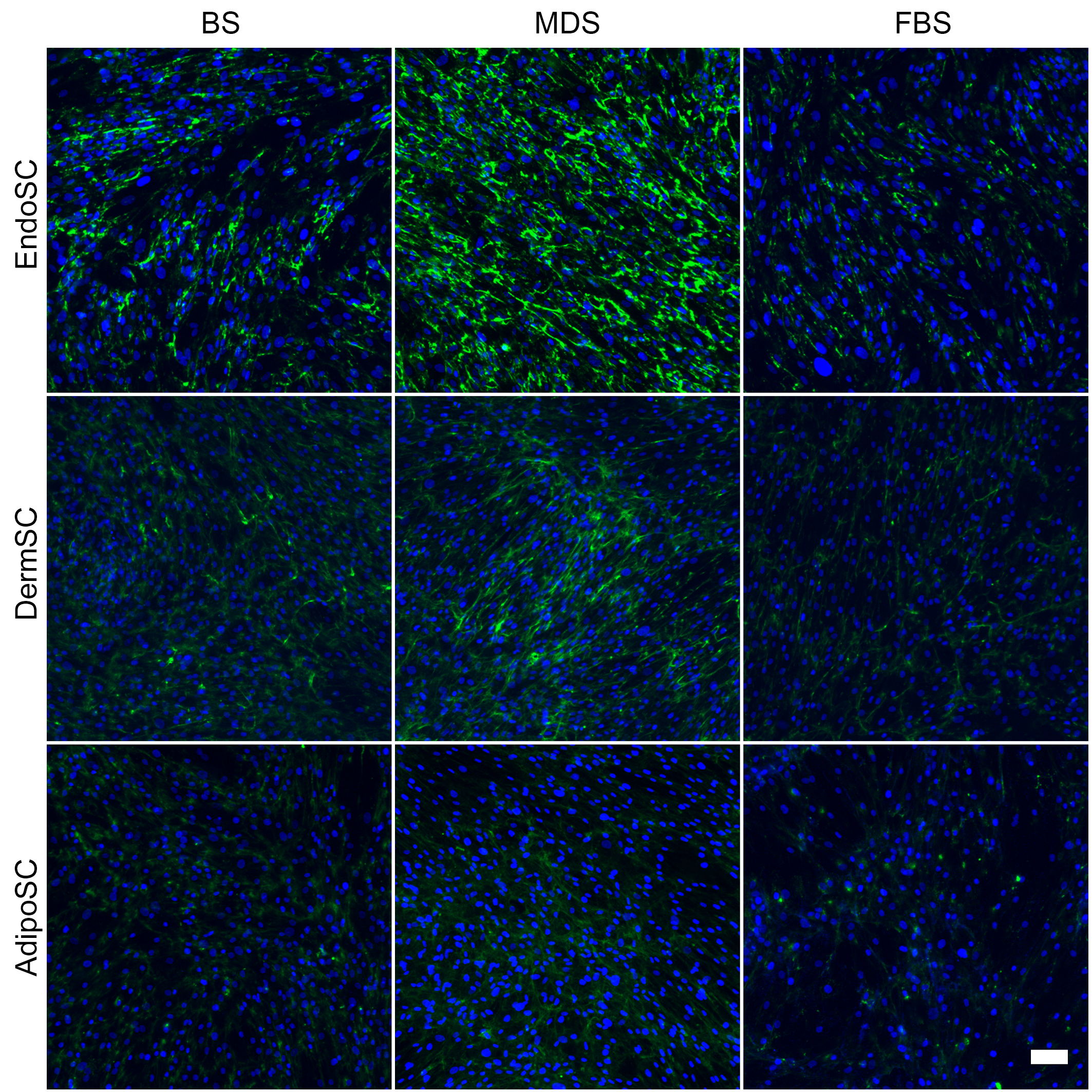

Supplement: Supplementary file 2 [file Image_1.TIF]

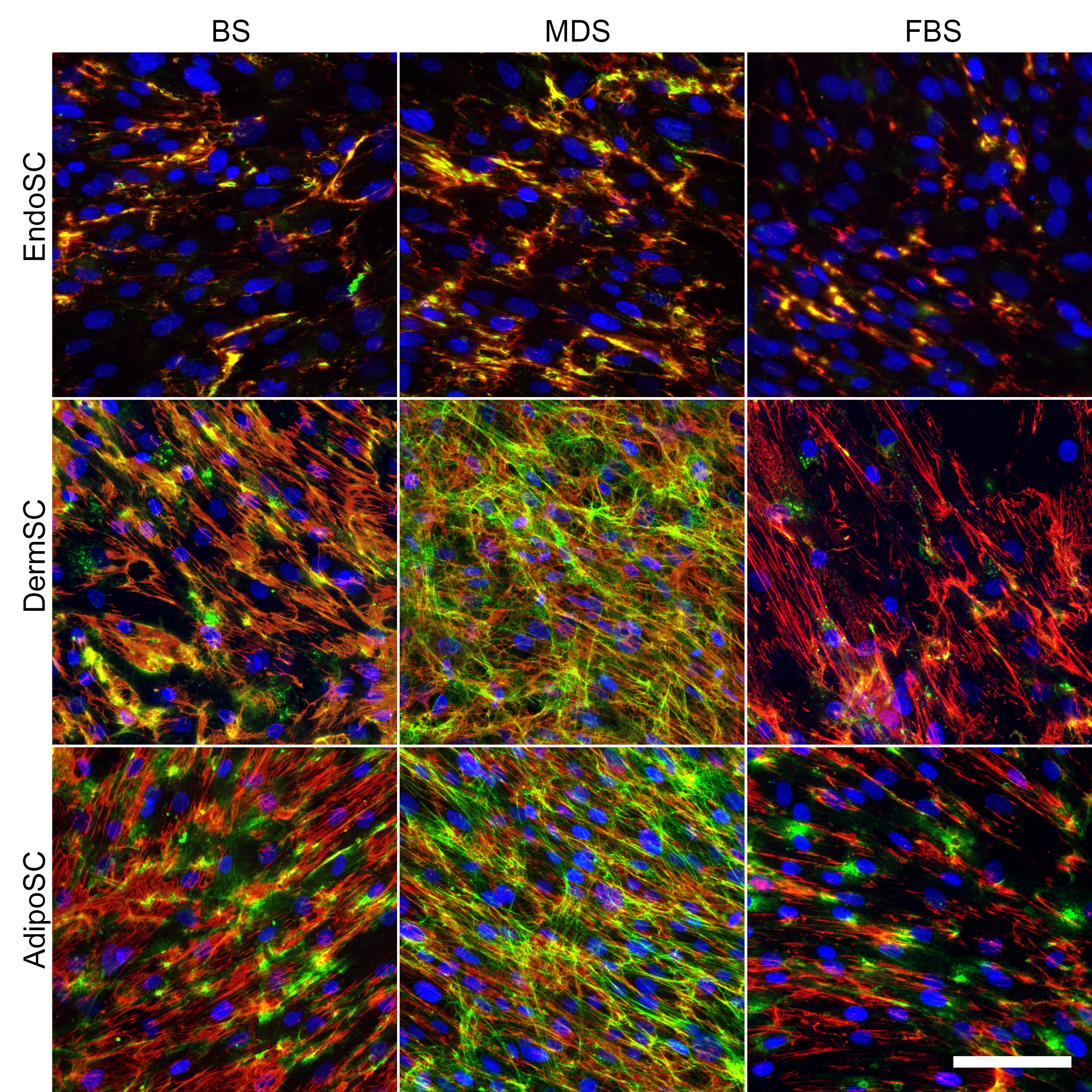

Supplement: Supplementary file 3 [file Image_2.TIF]
